# *De novo* transcriptome assembly and analysis of the freshwater araphid diatom *Fragilaria radians*, Lake Baikal

**DOI:** 10.1038/s41597-019-0191-6

**Published:** 2019-09-27

**Authors:** Yuri Pavlovich Galachyants, Yulia Robertovna Zakharova, Nadezda Antonovna Volokitina, Alexey Anatolyevich Morozov, Yelena Valentinovna Likhoshway, Mikhail Aleksandrovich Grachev

**Affiliations:** Limnological Institute, Siberian Branch of the Russian Academy of Sciences, 664033 3 Ulan-Batorskaya st., Irkutsk, Russia

**Keywords:** Next-generation sequencing, Sequence annotation

## Abstract

Diatoms are a group of eukaryotic microalgae populating almost all aquatic and wet environments. Their abundance and species diversity make these organisms significant contributors to biogeochemical cycles and important components of aquatic ecosystems. Although significant progress has been made in studies of Diatoms (Bacillariophyta) over the last two decades, since the spread of “omics” technologies, our current knowledge of the molecular processes and gene regulatory networks that facilitate environmental adaptation remain incomplete. Here, we present a transcriptome analysis of *Fragilaria radians* isolated from Lake Baikal. The resulting assembly contains 27,446 transcripts encoding 21,996 putative proteins. The transcriptome assembly and annotation were coupled with quantitative experiments to search for differentially expressed transcripts between (i) exponential growth phase and dark-acclimated cell cultures, and (ii) those changing expression level during the early response to light treatment in dark-acclimated cells. The availability of *F*. *radians* genome and transcriptome data provides the basis for future targeted studies of this species. Furthermore, our results extend taxonomic and environmental sampling of Bacillariophyta, opening new opportunities for comparative omics-driven surveys.

## Abstract (summary)

**Table:** 

Measurement(s)	transcription profiling assay
Technology Type(s)	RNA sequencing
Factor Type(s)	experimental condition • strain • response to light
Sample Characteristic - Organism	Fragilaria • Fragilaria radians
Sample Characteristic - Environment	freshwater lake biome
Sample Characteristic - Location	Lake Baikal

Machine-accessible metadata file describing the reported data: 10.6084/m9.figshare.9770687

## Background & Summary

Diatoms are unicellular photosynthetic aquatic eukaryotes that populate almost all aquatic and wet environments. Their abundance and diversity make these organisms important components of the biosphere. In particular, abundant marine phytoplankton species are responsible for about 20% of global primary production^1,2^. Diatoms also play an important role as primary producers forming the basis of grazer-based aquatic food webs in lacustrine and river ecosystems. Due to their abundance, diatoms are important contributors to the geochemical cycles of inorganic elements such as N, P, Si, which are concentrated from the water and can be sedimented after a short period of favorable conditions when the phytoplankton development occurs. Remarkable progress has been made in Bacillariophyta studies over the last two decades, due to the spread of “omics” technologies. The breakthrough in diatom “omics” was initiated by deciphering the complete nuclear genome sequences of *Thalassiosira pseudonana*^3^ and raphid pennate *Phaeodactylum tricornutum*^4^. Subsequently, several other diatom genomes have been sequenced^5–9^. *T*. *pseudonana* and *P*. *tricornutum* became emerging model species and quantitative gene transcription analyses were performed under various conditions to decipher the orchestrated changes in gene transcription patterns due to external factors^10–13^. For a number of diatom species, the transcriptomes were sequenced within the MMETSP survey^14^, enabling comparative genomic analyses to be performed at the broader taxonomic scale. Other studies used specific taxonomic sampling designs to apply molecular phylogenetic testing for specific evolutionary scenarios within the phylogenomic framework^15^.

Despite extensive genomic data acquisition in recent years, current knowledge of diatom molecular processes and gene networks providing physiological responses to changing environments remains incomplete. Genome sequencing and RNA-seq of new diatom species along with validation of particular metabolic pathways and regulation cascades may facilitate studies of diatom molecular biology.

The freshwater araphid diatom *F*. *radians* (Kützing) D.M. Williams & Round is a cosmopolitan planktonic diatom widely distributed in oligotrophic and mesotrophic water reservoirs across the northern hemisphere. The former name of this diatom, *Synedra acus* subsp. *radians* (Kützing) Skabitsch., was used in our previous studies to acknowledge the original taxonomic definition given by Sckabitchevskii in 1960 for *F*. *radians* in Lake Baikal. However, the genus *Synedra* was moved to *Fragilaria* by Willians and Round, 1987^16^ based on morphological features and the two species names, *F*. *radians* and *S*. *acus* subsp. *radians* are homotypic synonyms denoting the same species^17^. *Synedra*, *Fragilaria*, and *Ulnaria* genera are still under taxonomical revision. Particularly, *Fragilaria* and *Ulnaria* genera (Kützing) were redefined^18^, while species complex of *Ulnaria* genus is under further revision which includes species typification^19^ and novel species description^20–23^. *F*. *radians* is often observed as a dominant species in Lake Baikal^24^. This diatom is still the only freshwater species to which genome and transcriptome analyses have been applied. This species was used for analysis of conservative fragments of common and specific diatom genes such as the largest subunit of RNA-polymerase II (*rpb*1)^25^ and silicon transport protein (*sit*)^26^. The draft genome assembly of *F*. *radians* was published earlier^7^. The predicted amino acid sequences from the genes present in this genome assembly were used to reconstruct evolutionary history of several diatom genes such as aquaporins^27^, chitin synthase^28^ and actin-related proteins^29^, as well as in a broad-scale phylogenomic survey^30^. A silicon transporter gene was also sequenced from both DNA and cDNA (complementary DNA) of this species^31^. Here, we describe a *de novo* assembly of the *F*. *radians* transcriptome.

## Methods

### Culture growth conditions and preparation of total RNA

Two axenic strains, *F*. *radians* A6 and *F*. *radians* BK280, were isolated from spring water specimens sampled near Bolshye Koty settlement, Southern Baikal (N 51°53′35.83″, E 105°04′36.34″), using the technique described by Shislyannikov *et al*.^32^. Both strains are maintained in our culture collection at Limnological Institute, Irkutsk and are available upon request. To generate material for RNA sequencing, cells were grown in 2L Erlenmeyer flasks with DM medium^33^ at 16 °C under daylight (the average intensity was 17 μmol of photons per square meter per second). The light/dark cycle during cultivation was 17/7 hours. Once cultures reached the target density of ~30E3 cells/ml (Supplementary Fig. 1a), two flasks of strain A6 in the mid-day light phase of cultivation were centrifuged (3,500 g, 10 min) to collect cells, which were frozen in liquid nitrogen. These two biological replicates represented non-synchronized cultures in exponential growth, “NExp”. For strain BK280, six flasks of diatom culture at a density of ~30E3 cells/ml were dark-acclimated at 4 °C for 48 hours. Next, two flasks were immediately centrifuged and cell pellets were frozen in liquid nitrogen (dark-synchronized cultures, “DSLT-0”). The remaining four bottles which represented the dark-synchronized light-treated cultures (“DSLT-XX”), were placed to an incubation chamber at 4 °C and light intensity of 6 μmol of photons per squared meter per second, generated by Fluora L36W77 phytolamps (Osram). Two biological replicates were taken in parallel to collect and freeze cells after 20 and 40 min of culture exposure to light (Table 1). Subsequently, the frozen cell pellets ranging in wet weight between 100 and 200 mg were processed with an RNeasy Plant Kit (Qiagen) following the manufacturer’s protocol. Before freezing, a small aliquot of cell culture was taken from each pellet to analyse bacterial contamination using DAPI (4′,6-diamidino-2-phenylindole) staining followed by epifluorescence microscopy^32^. All samples except one were found to be axenic by means of DAPI staining (Table 1, Supplementary Fig. 1b).

**Table 1:** Description of samples used to acquire RNA-seq data.

Sample id	Sample name	Group	Strain	Cell divisions are synchronized	Biological replicate	DAPI test	Exposure to light, min	RIN at LIN	RIN at FGCZ	RNA concentration (ng/ul)	Raw reads* 10^6^
1	NExp1	Nexp	A6	No	1	passed	200	8.1	8.2	175	15.9
2	NExp2	Nexp	A6	No	2	passed	200	8.9	8.3	52	8.9
3	DSLT1-0	DSLT	280	Yes	1	passed	0	8.0	8.3	544	6.8
4	DSLT2-0	DSLT	280	Yes	2	passed	0	8.2	7.2	612	7.9
5	DSLT1-20	DSLT	280	Yes	1	passed	20	8.5	7.7	638	11.9
6	DSLT2-20	DSLT	280	Yes	2	passed	20	8.2	8.1	717	10.8
7	DSLT2-40	DSLT	280	Yes	2	passed	40	8.3	8.2	608	7.4
8	DSLT1-40	DSLT	280	Yes	1	not passed					

### Quality control of total RNA samples, library preparation and sequencing

The evaluation of RNA quantity and quality was performed spectrophotometrically by UV absorbance at 230/260/280 nm. Fragment lengths distribution analysis was performed using RNA 6000 Nano LabChip Kit for microcapillary electrophoresis and 2100 Bioanalyzer (Agilent, USA). The RIN (RNA Integrity Number) values of all total RNA samples were above 8. RNA samples were ethanol-precipitated, dried and sent to FGCZ (Functional Genomics Center, Zurich, Switzerland) for cDNA library preparation and sequencing. At FGCZ, samples were dissolved in 20 μl of molecular biology grade water and assayed with Agilent TapeStation to measure RIN again. The RIN (RNA integrity number) values were all above 7 prior to complementary DNA synthesis. Total RNA samples were used for preparation of cDNA-libraries according to standard Illumina protocol. This procedure included polyA-enrichment of total RNA followed by cDNA library generation with dUTP single-strand TrueSeq protocol. cDNA libraries were sequenced using Illumina HiSeq 2500 instrument and HiSeq SBS250 Kit V4. In total, seven cDNA libraries were sequenced at a half of HiSeq 2500 lane producing 7–15 millions of 125-bp paired-end sequencing reads per sample (Table 1). Raw sequencing reads are available at NCBI Sequence Read Archive^34^.

### *De novo* transcriptome assembly and evaluation

**Fig. 1:**
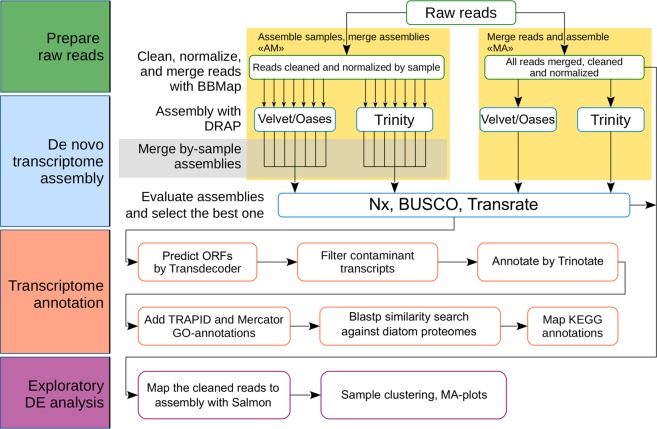
Flowchart of the bioinformatic analysis pipeline. Several *de novo* transcriptome assembly strategies were applied and evaluated to find the most optimal one. This optimal transcriptome assembly was obtained with “merge reads → assemble with Trinity” workflow (“MA-Trinity”) and was then subjected to annotation and secondary bioinformatics analyses.

**Fig. 2:**
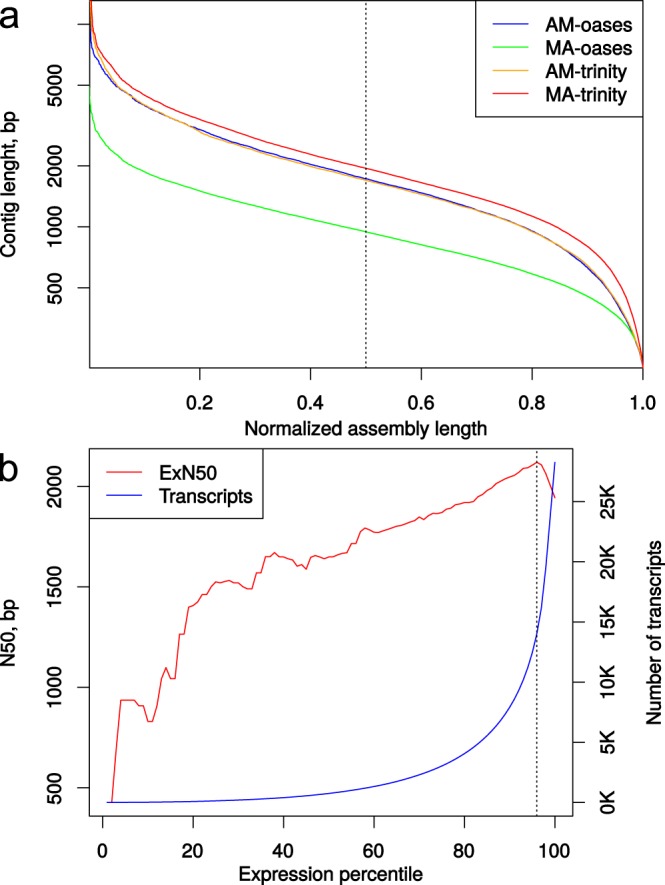
Transcriptome assembly results. Transcriptome assembly statistics reveal the best strategy to generate the high quality *de novo* transcriptome assembly. (**a**) Nx curves computed for RNA-seq assemblies. Vertical dotted line is drawn at 0.5 normalized assembly length. Colour of a curve encodes strategy used to generate the assembly (see Fig. 1 and Table 2 and Methods for more details). (**b**) N50 graph by expression percentiles plotted for the best assembly generated by “merge reads → assemble with Trinity” strategy. ExN50 – red line (y-scale on the left), number of transcripts – blue line (y-scale on the right). Vertical dotted line is drawn through the maximum of the ExN50 curve, showing that 14,009 transcripts are covered by 96% of reads and N50 of this assembly subset is equal to 2,120 bp.

Raw sequencing reads were filtered and normalized with BBMap v.37.01 package [https://github.com/BioInfoTools/BBMap]. Two workflows were examined to choose an optimal transcriptome assembly strategy (Fig. 1). We tested Trinity v.2.5.1^35^ and Velvet v.1.2.10/Oases v.0.2.09^36^ as DBG-assemblers (de Bruijn graph assemblers) incorporated into DRAP v.1.91^37^ pipeline. We also tested different variants of input data preparation to assemble the transcriptome: (i) feed the separate RNA-seq datasets to DRAP (runDrap) and merge these pre-assemblies with runMeta (assemble reads by samples → merge assemblies, “AM”); (ii) merge the sequencing reads together and assemble with runDrap (merge reads from all samples → assemble, “MA”). See Table 2, and data files {oases|trinity}_{AM|MA}.fa.gz^38^. For each assembly variant, DRAP report metrics were examined. Additionally, we analysed Nx metrics (Fig. 2a), the coding capacity of the assemblies using BUSCO v.3^39^ and the scores generated by re-mapping input reads using Transrate v.1.0.1^40^. The conventional Nx length statistics shows that at least x% of the assembled transcript nucleotides are found in contigs that are of at least Nx length. This means the higher Nx curve is, the more contiguous transcripts the assembly consist of (Fig. 2a). ExN50 statistics, which can be generated by Trinity accessory scripts [https://github.com/trinityrnaseq/trinityrnaseq/wiki/Transcriptome-Contig-Nx-and-ExN50-stats], seems more appropriate for transcriptome data^35^. In this case N50 is calculated for a subset of top highly expressed transcripts covered by x% of sample-wise normalized reads. For max(ExN50), the closer “x” value to the 100%, the better coverage of long transcripts in an assembly is. Usually, if the ExN50 has a maximum by “x” above 90%, the assembly is considered to provide a good coverage (Fig. 2b) and deeper sequencing will unlikely to produce for the higher quality assembly.

**Table 2:** Summary of assembly statistics generated by various pipelines.

Workflow*	Velvet/Oases		Trinity	
	1	2	1	2
**General assembly metrics**				
Lenght, Mbp	11.37	17.92	16.64	41.76
Number of transcripts	9,076	22,275	13,285	28,263
N50, bp	1,726	945	1,695	1,944
L50	2,099	6,446	3,112	6,840
**Transrate**				
Score	0.13	0.10	0.18	0.39
Optimal score	0.16	0.12	0.22	0.44
Optimal cutoff	0.26	0.24	0.29	0.35
Good contigs, %	79	80	80	87
BUSCO results**				
Complete	120	62	167	267
Fragmented	18	95	18	7
Missed	165	146	118	29

*(1) assemble samples → merge assemblies (AM); (2) merge reads → assemble (MA).

**BUSCO Eukarya database OrthoDB v.9, 303 busco genes.

### Transcriptome annotation

Transcriptome assembly annotation (Fig. 1) was performed with Trinotate v.3.1.1 pipeline [http://trinotate.github.io]. First, DRAP contigs were scanned with Transdecoder v.5.0.2 [http://transdecoder.github.io]. Options ‘–retain_[blastp|pfam]_hits’ were used at the prediction stage to decrease false-positive ORF (open reading frame) discovery. DRAP contigs were scanned by blastx against UniProt/Swiss-Prot release 2018_04^41^ database. Transdecoder-predicted ORFs were analysed with blastp against the NCBI NR (non-redundant genes) database to find potential contaminant sequences. A simple combination of rules was used to find contigs originating outside of the *F*. *radians* transcriptome. To fall into this category, the best blast hit from the Transdecoder-predicted ORF against NR had to (i) have identity > 90%, (ii) e-value < 1E-20, (iii) query coverage per subject > 40%, (iv) and taxonomic affiliation with lineages of primates, muridae or bovidae, as these groups were found to be the main sources of contamination. The filtered transcriptome assembly was used as input for the Trinotate package. Nucleotide sequences were analysed by blastx and the corresponding ORFs scanned by blastp against the UniProt/Swiss-Prot database. ORFs were searched with (i) hmmscan v.3.1b2 [http://hmmer.org] against Pfam-A release 31.0^42^, (ii) TmHMM v.2.0c^43^, and (iii) SignalP v.4.1^44^. Nucleotide sequences were analysed by RNAmmer^45^. Results of these analyses were loaded into a local database and merged using Trinotate.

For ORFs of filtered transcriptome contigs we then extended the Trinotate-derived GO (Gene Ontology) annotations by those from TRAPID^46^ and Mercator^47^ web-based annotation pipelines. In the TRAPID analysis, PLAZA 2.5 was used as a reference database. Mercator analyses included all available reference databases except for InterProScan. For each ORF, TRAPID- and Mercator-generated annotations were compared with Trinotate results and new GO terms were aggregated with the existing ones (Supplementary Fig. 2a). To measure TRAPID/Mercator similarity with Trinotate annotations, the corresponding sets of GO terms were compared using GOSemSim R package^48^. For each transcript, Wang, Lee, and Jiang similarity indices aggregated by “Best-Match-Average” algorithm were computed between Trinotate and TRAPID/Mercator sets of GO terms (Supplementary Fig. 2b).

To assess convergence of the transcriptome assembly with *F*. *radians* draft genome sequence^7^ and gene sets of closely related diatom species, we performed blast searches against (i) *F*. *radians* draft assembly genomic scaffolds^49^, and (ii) filtered gene models of *T*. *pseudonana*^3^ (NCBI Genome ID GCF_000149405.2) and *P*. *tricornutum*^4^ (NCBI Genome ID GCF_000150955.2). NCBI blast+ v. 2.2.28^50^ was used for all blast searches. A web-based OrthoMCL^51^ analysis was performed to assign transcriptome ORFs to orthologous groups in the OrthoMCL database, release 5 (Table 3, File_1_orthoGroups.by_group^38^).

**Table 3:** OrthoMCL statistics.

Species	OrthoMCL Taxonomic category	Gene set type	Number of			
			Input sequences	Sequences assigned by OrthoMCL	OrthoMCL Groups	BBH with *F*. radians*
*Fragilaria radians*	VIRI	transcriptome	22 813	19 339	7 893	—
*Thalassiosira pseudonana*	VIRI	genome	11 776	8 775	5 607	9 474
*Phytophthora ramorum*	OEUK	genome	15 743	13 493	5 730	849
*Nematostella vectensis*	META	genome	27 273	21 362	11 201	423
*Arabidopsis thaliana*	VIRI	genome	33 200	29 730	12 546	370
*Oryza sativa*	VIRI	genome	26 777	20 655	11 024	314

*Number of proteins having best BLAST hits with *F*. *radians* transcriptome ORFs.

To further validate *F*. *radians* transcriptome annotations, we compared them with annotations of the closest orthologous genes belonging to diatoms and other protists. First, *F*. *radians* transcripts encoding ORFs were subjected to blastp analysis against proteins of *T*. *pseudonana* (NCBI Genome ID GCF_000149405.2), *P*. *tricornutum* (NCBI Genome ID GCF_000150955.2), and *F*. *cylindrus* (NCBI Genome ID GCA_001750085.1). For *F*. *radians* transcripts having significant hits (e-value < 1E-20, query coverage per subject > 50%), annotations from diatom genes were applied. Next, the presence of annotations was checked for transcripts with significant blastp-hits against NCBI NR. Hits with a KEGG orthogroup assigned and belonging to protists were used to confirm gene descriptions of *F*. *radians* transcripts. For the rest of Trinotate transcripts with a KEGG (Kyoto Encyclopedia of Genes and Genomes) orthologous group, annotations were left unaltered, and a specific tag was assigned to show the transcript has no similar sequences within the protists. Assembled and annotated sequences are available at DDBJ/EMBL/GenBank^52^. Annotation is also available as tab-delimited table at figshare^38^ (File_2_Trinotate_report.csv.zip and Table_S2.xlsx).

To visualise the overall pattern of functions assigned to annotated transcriptome and its subsets, a projection of GO terms into GO-slim space was performed using R package GSEABase^53^. GO terms were mapped and for those exceeding 1% were bar plots were generated (Supplementary Figs 2c and 3b).

### Differential gene expression analysis

**Fig. 3:**
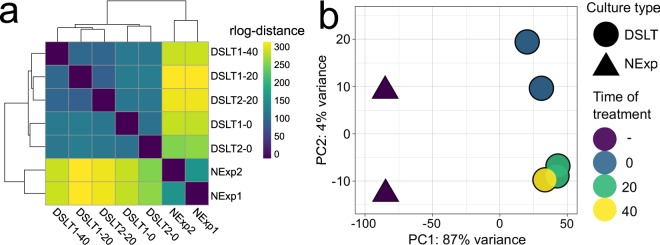
Exploratory analysis of RNA-seq samples similarity. Clustering and principal component analysis show that samples are grouping by biological replicates and experimental design. There exists a sharp difference between NExp and DSLT groups while the samples split into two subgroups inside DSLT depending on whether a culture was exposed to light. The sample pairwise distances were computed on a matrix of regularized log-transformed transcript counts. (**a**) Heat map of sample distances. Hierarchical sample clustering is presented on the top and left sides. (**b**) Principal component analysis of RNA-seq samples.

Filtered sequencing reads were re-mapped to *F*. *radians* DRAP transcriptome assembly using Salmon v.0.9.1^54^ and analysed with DESeq2 R package^55^. Raw read counts were filtered to exclude orphan transcripts. A regularized log-transformed matrix of transcript expression counts was used for hierarchical clustering of samples by computing a matrix of pairwise sample distances (Fig. 3a), and for ordination of samples by principal component analysis (PCoA) (Fig. 3b). An adaptive *t* shrinkage estimator^56^ was used for ranking and visualisation of log-fold changes (log_2_FC, LFC) of differentially expressed (DE) genes (Supplementary Fig. 4, Table_S3.xlsx^38^).

## Data Records

Raw sequencing reads are available at NCBI Sequence Read Archive (SRA) under accession SRP156385^34^. Transcriptome assemblies presented in Table 2 ({oases|trinity}_{AM|MA}.fa.gz) and accessory data associated with the assembly used for annotation and further analysis were uploaded to figshare as a file set^38^. Draft genome sequence of *F*. *radians* is available at European Nucleotide Archive (ENA) under accession CAAAJI010000000^49^. The Transcriptome Shotgun Assembly project has been deposited at DDBJ/EMBL/GenBank under accession GGVJ00000000^52^. The version described in this paper is the first version, GGVJ01000000.

## Technical Validation

### Transcriptome assembly

For transcriptome analysis, strand-oriented cDNA libraries generated from non-normalized polyA-enriched RNA were sequenced. In total, 69.5 million 125 bp paired-end Illumina reads were obtained from seven cDNA libraries (Table 1).

As determined from the assembly evaluation (Fig. 1), the best strategy for assembling *F*. *radians* RNA-seq data was the DRAP/Trinity workflow applied to merged, cleaned, and normalized raw sequencing reads (merge reads → assemble with Trinity, “MA-Trinity”). Compared to this assembly strategy, other options for input data and tools produced inferior assemblies by one or more key QC parameters. The “MA-Trinity” strategy resulted in the longest (Table 2) and most contiguous (Fig. 2a) transcriptome assembly which has the highest Transrate score and contains the greatest number of complete eukaryotic BUSCO genes. According to the BUSCO analysis, only 29 (9.5%) core eukaryotic genes were missing from the “MA-Trinity” assembly.

This DRAP assembly containing 28,263 contigs was annotated using a pipeline described in Methods (Fig. 1). All 22,813 ORFs discovered in the DRAP assembly by Transdecoder had a one-to-one relationship with the contigs. An ORF sequence scan against NR revealed 817 contaminant contigs and one chimeric sequence. Principally, contaminant sequences fell into the category of low-expressed transcripts. Nine of these 817 contigs fell into the 86-94-percentile expression level range, and 808 were expressed below the 95^th^ percentile (Table_S1.xlsx^38^ and Fig. 2b). Contaminant sequences were removed from the assembly. Contig CL748Contig1_1 appeared to be chimeric. The 3′-containing ORF had low RNA-seq read coverage and was highly similar to human 18S rRNA. Conversely, the 5′-end of this contig appeared to be highly transcribed, falling into the 30th percentile expression bin (File_3_salmon.isoform.TMM.EXPR.matrix.E-inputs.zip)^38^ and had no hits against NR. This contig was manually clipped by 3′-end for downstream analyses. The filtered assembly was used for annotation and DE analyses.

### Properties of the annotated transcriptome gene set

The final set of assembled and filtered contigs contained 27,446 transcripts encoding 21,996 ORFs. Recovery of BUSCO genes within the protein-coding set was slightly lower than at the transcript level (Table 2), with 54 (18%) single-copy eukaryotic orthologs missing. The Trinotate pipeline recovered a considerable number of functional gene annotations. More than 17,000 protein sequences had robust best blast hits against NCBI NR and more than 10,000 ORFs produced significant hits against UniProt/Swiss-Prot (Table 4). In total, more than 19,000 GO terms were mapped to protein-coding transcripts from UniProt/Swiss-Prot and Pfam-A similarity hits. More than 18,000 additional non-redundant GO terms were mapped to 6,314 *F*. *radians* genes from TRAPID or Mercator annotations. Generally, updated annotations originated from TRAPID results (Supplementary Fig. 2a). Importantly, the majority of new annotations had a semantic similarity of <0.75 with existing Trinotate annotations (Supplementary Fig. 2b) and overlap in the GO-slim space (Supplementary Fig. 2c). This suggests that the TRAPID/Mercator pipeline extended standard Trinotate results with new functional descriptions from manually-curated photosyntetic- and protist-related databases.

**Table 4:** Summary of functional annotation results.

	Unique	Total
**Trinotate annotations**		
BBxH* against Uniprot/Sprot	12 190	12 383
BBpH** against Uniprot/Sprot	10 218	10 686
Pfam hit	11 227	11 817
BBxH against NR	20 556	20 637
BBpH against NR	17 198	17 776
KEGGs mapped	6 286	10 783
EggNOGs mapped	2 717	9 241
GOs mapped from Uniprot/Sprot hits	5 919	11 925
GOs mapped from Pfam hits	1 224	7 337
TmHMM	5 342	5 747
SignalP	2 235	3 368
RNAMMER	18	18
**Additional annotations**		
Genes updated by TRAPID/Mercator	—	6 314
GOs mapped by TRAPID/Mercator	—	18 622
OrthoMCL group assigned	7 893	19 339

*BBxH – best blastx hit.

**BBpH – best blastp hit.

Annotated transcriptome ORFs were mostly in agreement with the *F*. *radians* draft genome assembly^7^. At the nucleotide sequence level, 21,132 of 27,446 transcripts including 17,634 of 21,996 ORFs had good blastn matches (e-value < 1E-50, query coverage per subject > 90%) with scaffolds from the *F*. *radians* draft genome. Similarly, 17,916 of 21,996 *F*. *radians* ORFs including 11,065 of the 13,526 annotated ORFs had good tblastn matches (the same settings as above) against *F*. *radians* genome scaffolds. Furthermore, 10,966 *F*. *radians* ORFs revealed good blastp matches (e-value < 1E-10, query coverage per subject > 70%) against filtered gene models of *T*. *pseudonana* and *P*. *tricornutum*, with 8,563 of them being annotated by Trinotate and 7,172 having good tblastn matches against *F*. *radians* genome scaffolds.

A procedure aiming to search and validate *F*. *radians* transcript ORF annotations generated a list of 8,710 transcripts with meaningful descriptions transferred from NCBI NR or UniProt/Swiss-Prot blastp hits. Annotations for 8,254 were borrowed from genes of diatoms or protists and 7,135 transcripts were assigned to KEGG orthogroups (Table_S2.xlsx)^38^.

### Overall transcript expression patterns and clustering of RNA-seq samples

The transcriptome assembly had good sequencing read coverage. The transcript expression analysis showed that about half of the assembled transcripts are covered by 96% of reads (see the intercept of vertical dotted line in Fig. 2b with red and blue curves). Assembly E90N50 was 2,047 bp, which is similar to assembly N50 (1,944 bp), suggesting the transcript coverage had a small influence on transcript length in a subset of low-expressed sequences.

The sample clustering revealed by exploratory analyses of transcript expression levels generally followed the experimental design with two biological replicates corresponding to NExp cultures clustering separately from DSLT samples (Fig. 3a). The majority (87%) of explained variance was distributed along the first principal component in the analysis of all-against-all sample distances (Fig. 3b), suggesting sharp difference between NExp/DSLT states and less pronounced but significant changes between DSLT dark/light samples.

As the difference between NExp and DSLT transcription patterns was pronounced, the log_2_ fold-change threshold for DE-analysis was set to 1, i.e. we were looking for transcripts with at least a two-fold change in expression between NExp and DSLT conditions. For the NExp/DSLT comparison, there were 5,959 DE transcripts with *s* < 0.005 (Supplementary Fig. 4, Table_S3.xlsx)^38^, of which 3,215 had GO terms. For the analysis of DE transcripts between cells incubated in darkness and those exposed to light after a period of darkness, we used smaller log_2_ fold-change threshold. When LFC was set to 0.585 (i.e., transcripts were required to have at least 1.5-fold expression level difference between dark and light conditions), there are 858 DE transcripts (Supplementary Fig. 4, Table_S3.xlsx)^38^ in the dark/light comparison (*s* < 0.05) with 406 transcripts having GO terms.

Importantly, EggNOG transcript annotations^57^ mapped to COGs (Clusters of Orthologuous Groups) [https://www.ncbi.nlm.nih.gov/COG] revealed a spectrum of functional categories of genes transcribed under different conditions of cultivation similar to that of the whole-transcriptome assembly (Supplementary Fig. 3a). Additionally, distribution of GO terms for transcripts differentially expressed in these two contrasts shows similar pattern in GO-slim space (Supplementary Fig. 3b, see GO-slim terms shared between two contrasts). This implies that the “global” pattern of expression changes affects all COG categories and high-level nodes of Gene Ontology database irrespective of culture growth conditions. However, there are several high-level GO-slim terms that absent in one of two analysed contrasts. Particularly, NExp/DSLT subset alone has translation, cellular amino acid metabolic process, ribosome biogenesis, structural molecule activity, and transferring glycosyl groups activity GO terms (Supplementary Fig. 3b). On the other hand, carbohydrate metabolic process, vesicle-mediated transport and extracellular region GO terms are only presented in DSLT dark/light contrast.

## Usage Notes

### Properties of *de novo* transcriptome assembly of *F*. *radians*

The transcriptome was assembled from shotgun cDNA libraries of two axenic *F*. *radians* strains sampled from Lake Baikal. In addition to the findings presented herein, the experimental design has two important implications for future use of the raw data. First, using both fluorescent microscopy and bioinformatics methods, the assembly was confirmed to contain no significant bacterial contamination. This feature is especially valuable for many downstream bioinformatics applications including experimental validation of transcript expression, reconstruction of single gene phylogenies, quantitative RNA-seq analyses, comparative genomics/transcriptomics, and phylogenomic surveys. Second, as two *F*. *radians* strains were used for assembly, these data can hypothetically be used to search for single nucleotide polymorphisms (SNPs) in transcriptionally active genome regions encoding specific genes. Once mapped to a genome sequence, transcript- and SNP-related data may boost functional annotation of specific genes.

The essential assembly parameters such as contiguity, coverage, and completeness (Table 2 and Fig. 2a) were optimized using RNA-seq data and the final assembly is seemingly a relatively fair representation of the *F*. *radians* transcriptome (Fig. 2b) with respect to both repertoire of transcribed sequences and correspondence of recovered transcripts to those synthesized *in vivo*. It is not possible to guarantee that the assembly pipeline used will produce good results for other RNA-seq datasets. However, during RNA-seq data processing in *de novo* transcriptome projects, we recommend optimizing the assembly strategy by tweaking several pivot parameters/steps such as (i) data filtering and normalization methods, (ii) merging input data from different libraries or generation of separate DBG assemblies to be merged later in the pipeline, and (iii) the DBG assembler used.

Thus, we tested *de novo* transcriptome assembly strategies to choose an optimal assembly variant, which was subsequently used for annotation. In total, the resulting assembly contains 27,446 transcripts encoding 21,996 open reading frames. About a half of the transcriptome ORFs had good matches to UniProt/Swiss-Prot sequences, which enabled automatic annotation (Table 4). Furthermore, the amino acid sequence comparison using blastp revealed 50% (10,966) of the ORFs in the *F*. *radians* transcriptome to be highly similar to either *T*. *pseudonana* or *P*. *tricornutum* filtered gene models with 39% (8,563 out of 21,996) having functional annotations generated by the Trinotate pipeline. Importantly, the convergence of transcriptome ORFs with *F*. *radians* draft genome sequence^7^ was good but not complete. BLAST comparison of *F*. *radians* transcriptome with the draft genome sequence revealed that 77% of transcripts (21,140 out of 27,446) have good blastn matches. Similarly, robust tblastn hits were found in 81% (17,916 out of 21,996) of the transcriptome ORFs.

We suggest that misassemblies within long genomic repeats is a major source of discrepancies between *F*. *radians* genome and transcriptome sequences^7,8,31,58^. These inconsistencies can hardly be resolved without additional sequencing and assembly efforts.

Almost a half of the *F*. *radians* ORFs (10,661 of 21,966) had no associated GO terms, with 7,172 having hits against the NR database but missing matches in UniProt/Swiss-Prot and Pfam-A. This fact emphasizes that a considerable number of unannotated transcriptome sequences possesses good similarity with poorly annotated genes from NCBI NR such as diatom- or heterokont-specific hypothetical proteins and conservative proteins with unknown function. Thus, a significant part of the *F*. *radians* transcriptome is suggested to produce conservative proteins of unknown function.

Poorly characterized proteins, along with better-annotated sequences, can be used for ortholog/paralog classification by unsupervised clustering methods. The OrthoMCL analysis clearly shows that the majority of *F*. *radians* transcriptome ORFs can be assigned to gene families (Table 3 and File_1_orthoGroups.by_group.zip)^38^. Upon gene family assignment and finding the orthologous/paralogous sequences, it seems reasonable to spend additional time optimizing the clustering procedure, as the commonly available web-based and standalone tools lack specificity in non-model organisms and may produce noisy results when applied directly to diatom-related datasets. For example, the OrthoDB database^59^ only includes sequences from two model diatoms, *T*. *pseudonana* and *P*. *tricornutum*, and OrthoMCL^51^ web server contains data on a single diatom species, *T*. *pseudonana*. Other orthology-centric databases (EggNOG, Ensembl Compara) do not include any diatoms at all. While *T*. *pseudonana* and *P*. *tricornutum*, compared to other diatom species, are undoubtedly best-studied by “omics”-approaches, their predicted proteomes are possibly not sufficient to capture the complexity of diatom gene families due to huge genetic diversity observed within this taxon.

### Transcription patterns in dark-acclimated cells and during the early response to light exposure

Diatoms have been found to survive in complete darkness for long periods^60^. Several species form quiescent cells, which begin vegetation in response to light exposure after several years in the dark^61^. Quiescent cells appear morphologically similar to normal vegetative cells but can be different in physiology and ultrastructure^11^. Quiescent diatom cells have low metabolic rates and condensed cytoplasm with chloroplasts located at the center of the cell. Some time after returning to conditions appropriate for growth and division, the internal cell structure reverts to its normal state and the cell resumes vegetation as usual^11,61–66^. The lag-time to exponential growth depends on the diatom species and the length of dark period^64^. *P*. *tricornutum* has been reported to survive in complete darkness for up to six months^60^. When *P*. *tricornutum* cultures are subjected to prolonged dark-acclimation, cells pause at the G1 phase checkpoint^67^. In a G1-arrested dark-acclimated *P*. *tricornutum* culture, chloroplasts begin to divide after five hours and cell divisions have been observed after 8–12 hours of re-exposure to light, respectively^68,69^.

### Transcript expression patterns in exponential growth cultures

Total RNA was isolated from non-synchronized *F*. *radians* cells within steady-state exponential growth five hours after the light exposure. The dark/light cycle impacts cell cycle synchronization in diatoms; however, the degree of synchronization and the prevalent cell cycle stage differ between diatom species. Cell division has been reported to generally take place during dark and dusk periods in *T*. *pseudonana*^70^ cultures, while the daylight phase is used to replenish energy and metabolic resources. Light/dark cell cycle synchronization has been observed in *P*. *tricornutum*^71^ but was weaker than in *T*. *pseudonana*, not exceeding 20% of simultaneously dividing cells^12^. Interestingly, a good synchronization effect has been reported for *Navicula pelliculosa*^72,73^. The specific procedure (24:24 hours of light:dark cultivation followed by extensive light exposure) was shown to double the number of diatom cells within a three-hour time frame, suggesting *N*. *pelliculosa* cells were arrested at G2/M stage.

As the NExp *F*. *radians* cultures were harvested in the mid-day light phase, their transcription pattern was characterized by activated photosynthesis and protein biosynthesis processes typical of cells in exponential growth. Synchronization of the cell cycle is only partial and transcription patterns are hypothesized to be proportionally averaged across the cell states present in a culture. Thus, RNA-seq is expected to capture “smoothed” transcript expression levels.

The availability of *F*. *radians* transcriptome data provides a foundation for future detailed single-gene-targeted studies. Here, an RNA-seq approach was used to assemble the *F*. *radians* transcriptome and to compare transcriptional patterns for cells under different culture conditions. At the genome level, the transcriptome data will help us to justify, filter, and update annotations of gene models. Furthermore, these results extend taxonomic and environmental sampling of Bacillariophyta, opening new opportunities for comparative-“omics”-driven surveys.

## Supplementary information

Supplementary Information.

## Data Availability

The specific commands used to analyse RNA-seq data and to draw the article figures are available at https://github.com/yuragal/fradians-rnaseq.
