# Supplementary material for: De novo transcriptome assembly and analysis of the freshwater araphid diatom Fragilaria radians, Lake Baikal
Source: Sci Data. 2019 Sep 27;6:183. doi: 10.1038/s41597-019-0191-6 (PMC6765018; doi:10.1038/s41597-019-0191-6)
Supplement: Supplementary file 1 — Supplementary Information. [file 41597_2019_191_MOESM1_ESM.pdf]

Content:

Supplementary Figure 1 – page 2
Supplementary Figure 2 – page 3
Supplementary Figure 3 – page 4
Supplementary Figure 4 – page 5

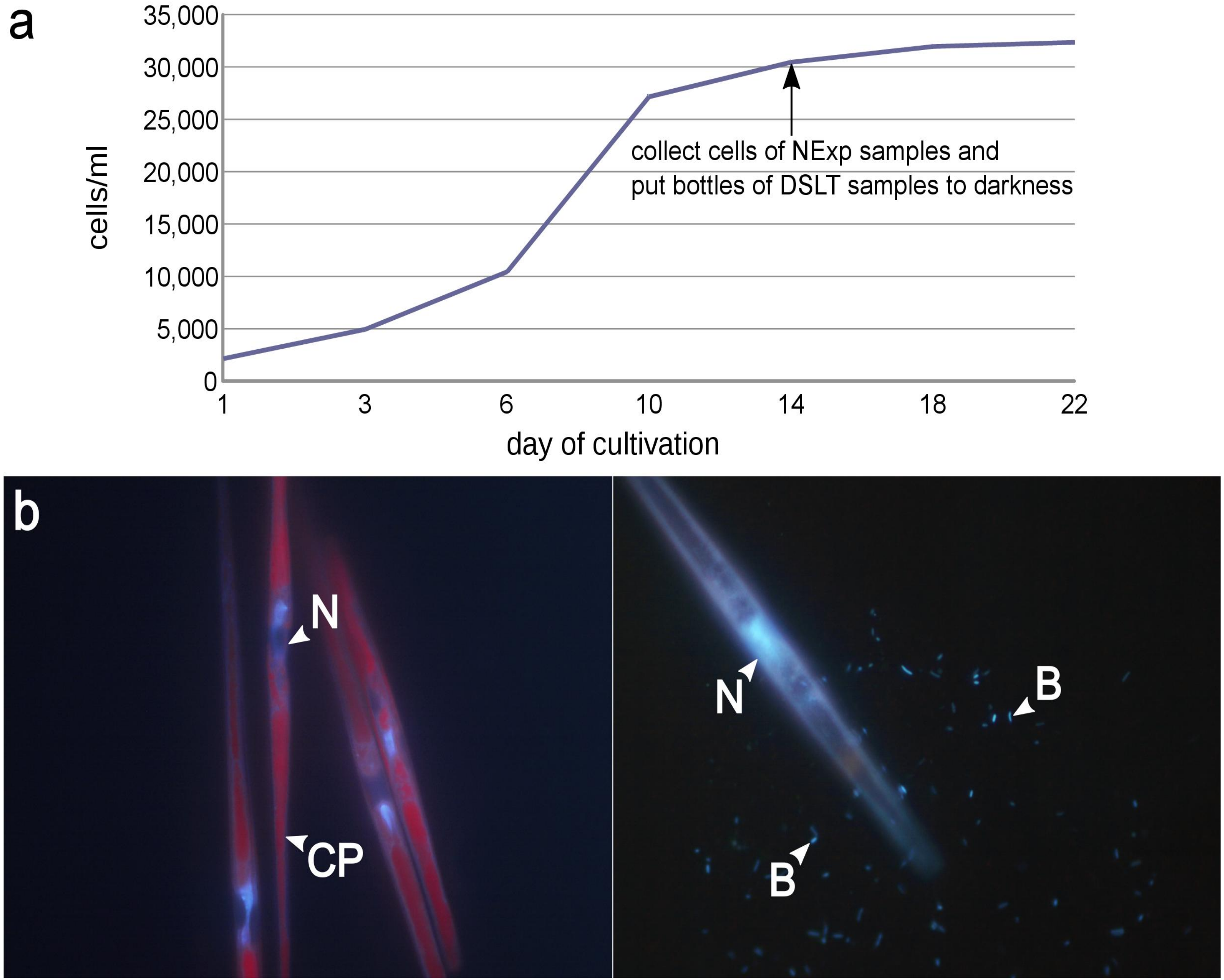

Figure S1. *Fragilaria radians* cultivation and epifluorescent microscopy.

[a] Growth curve of *F. radians* BK280. *F. radians* A6 strain has the same growth dynamics. [b] DAPI-staining: axenic culture, sample DSTL2-0 (on the left) and culture contaminated with bacteria, sample DSTL1-40 (on the right). N and CP – nucleus and chloroplast of *F. radians* cell; B – bacterial cell.

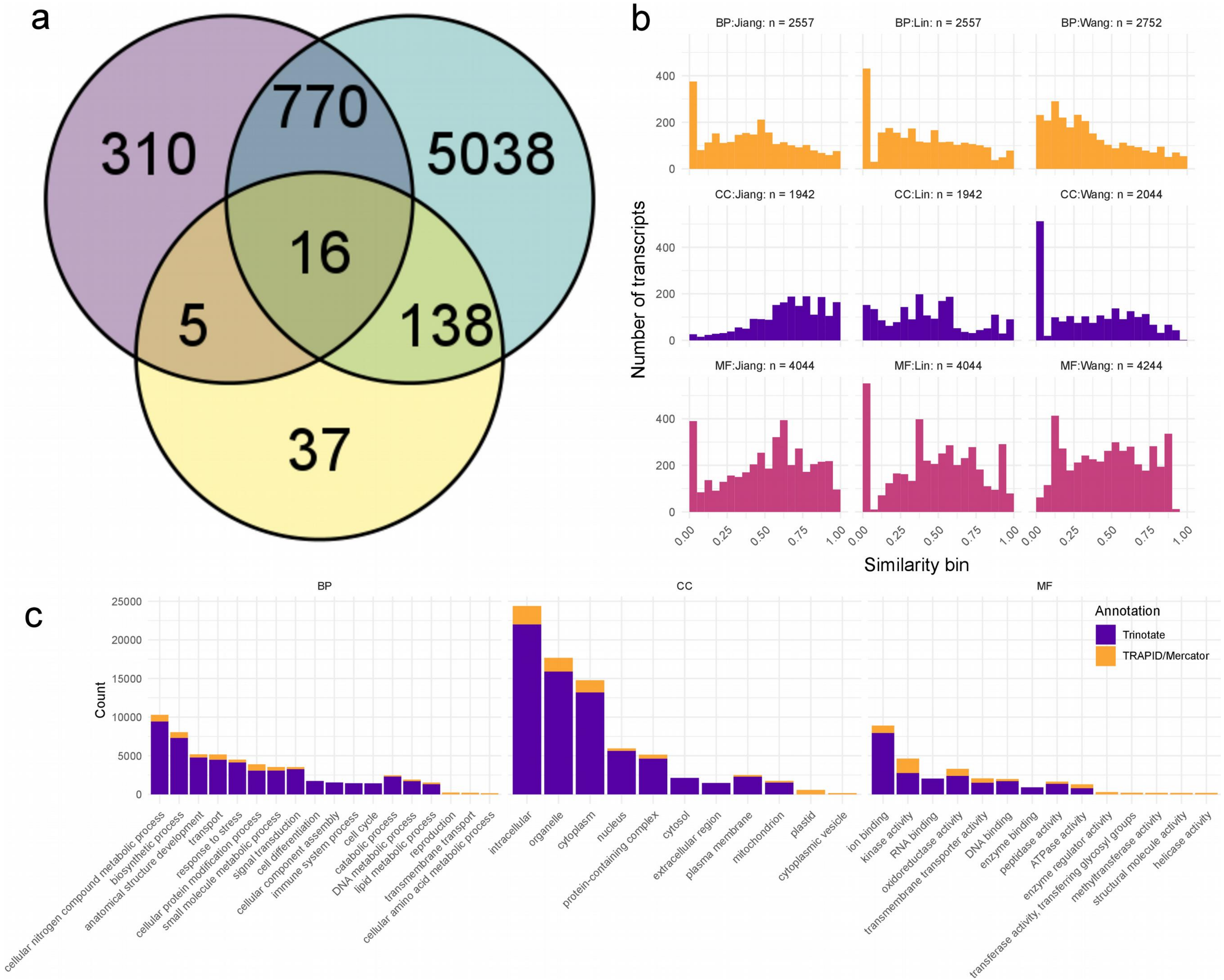

Figure S2. Transcriptome annotation results.

TRAPID/Mercator pipeline extended standard Trinotate results with new reasonable functional descriptions. [a] Comparison of GO terms added to Trinotate results by Mercator (violet) and TRAPID (cyan). Only GO terms absent in Trinotate annotations are shown. Yellow – GO terms shared by TRAPID and Mercator annotations. [b] Frequency of semantic similarity scores calculated for transcripts with updated annotations. Annotations generated by Trinotate were transcript-wise compared with those from TRAPID/Mercator pipelines by three semantic similarity indices Wang, Lee, and Jiang. Number of transcripts used for comparison is denoted in facet title. BP, MF and CC are the three high-level gene ontology terms: “biological process”, “molecular function”, and “cellular component”. [c] Top GO-slim terms from Trinotate (violet) and those added from TRAPID/Mercator (yellow). Included are GO-slim terms with frequency above 1% within a source subset. BP, MF and CC are as in Figure S2b.

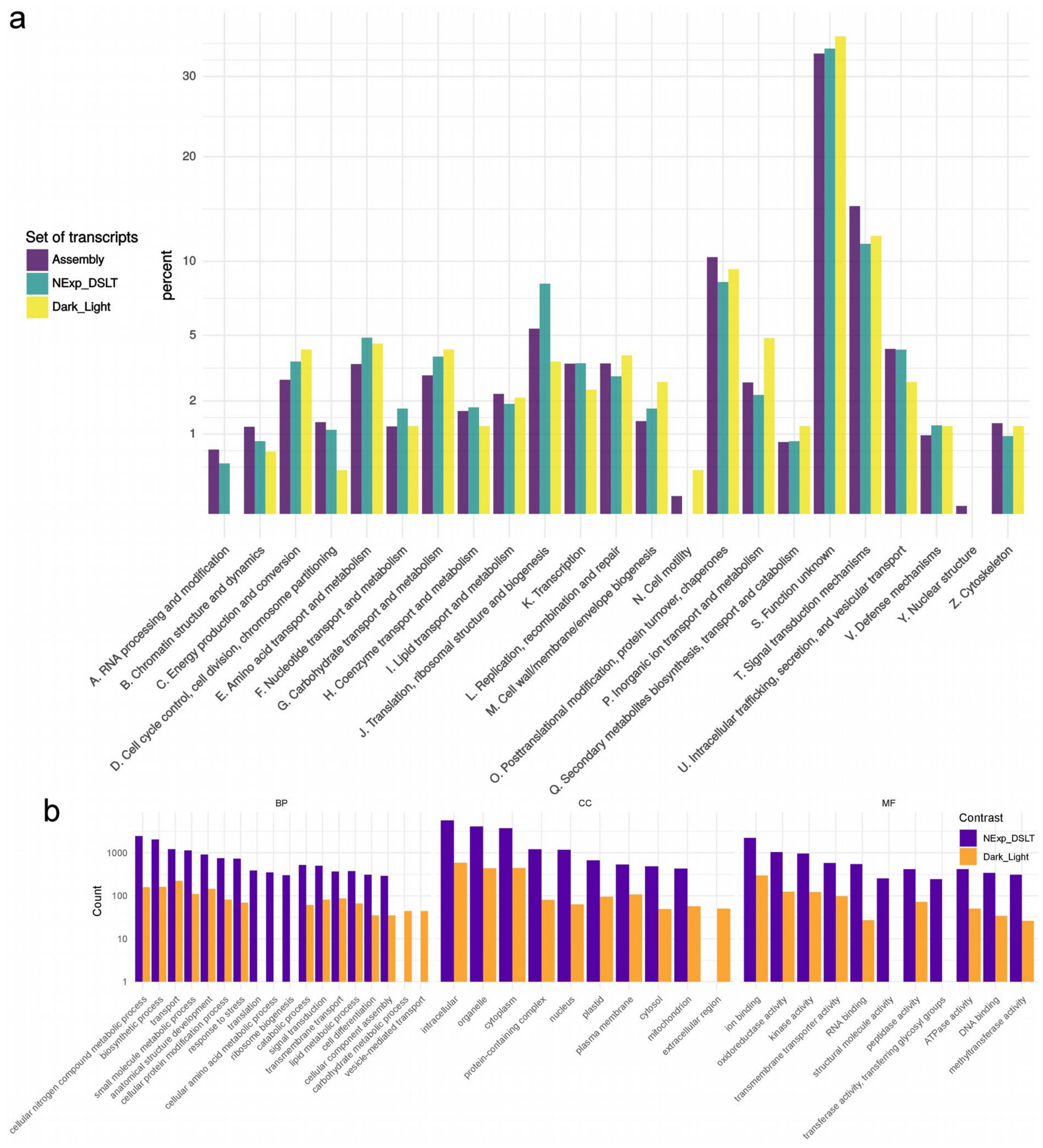

Figure S3. Functional annotation of differentially expressed transcripts.

Distribution of differentially expressed transcripts by COG-categories and GO-slim terms is similar to those of the whole-transcriptome assembly. [a] Distribution of transcripts by COG functional categories summarized from EggNOG transcriptome annotations. All annotated transcripts – violet bars. Subsets of transcripts differentially expressed in NExp/DSLT and DSLT dark/light contrasts are cyan and yellow bars. [b] Top GO terms of transcript subsets differentially expressed in NExp/DSLT (violet) and dark/light (yellow) contrasts. Included are GO-slim terms with frequency above 1% within a source subset. BP, MF and CC are as in Figure S2b.

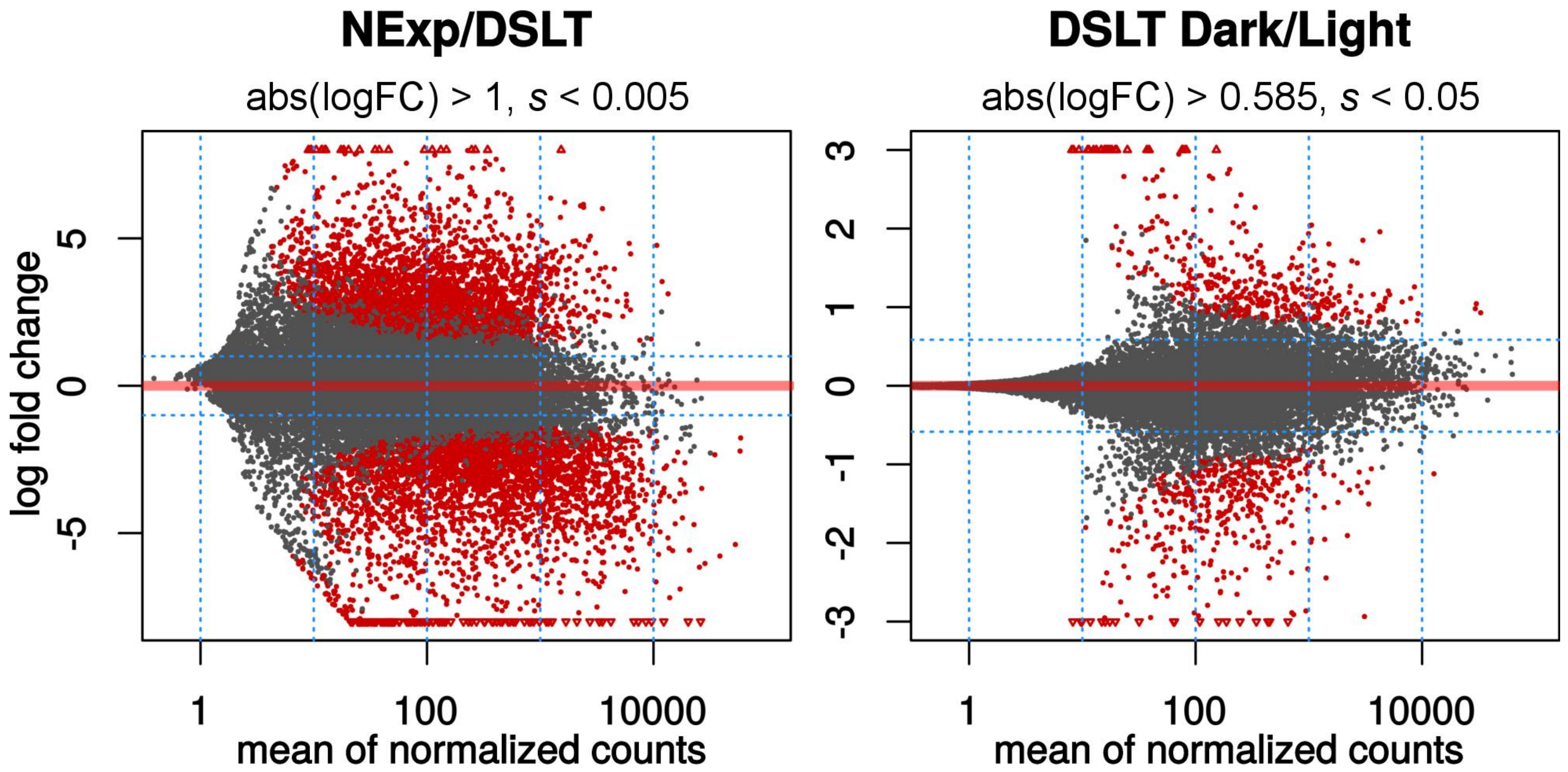

Figure S4. Mean-average plot of the transcript expression levels.

NExp/DSLT contrast is shown of the left and DSLT dark/light contrast – on the right. Differentially expressed transcripts (red dots) are identified by Log_2FC and *s*-value as shown in the facet subtitle. Other transcripts – grey dots.
